# A Hybrid CNN-Transformer Model for Predicting N Staging and Survival in Non-Small Cell Lung Cancer Patients Based on CT-Scan

**DOI:** 10.3390/tomography10100123

**Published:** 2024-10-10

**Authors:** Lingfei Wang, Chenghao Zhang, Jin Li

**Affiliations:** College of Intelligent Systems Science and Engineering, Harbin Engineering University, Harbin 150001, China; wanglingfei@hrbeu.edu.cn (L.W.); 910381219@hrbeu.edu.cn (C.Z.)

**Keywords:** non-small cell lung cancer, N-staging prediction, survival analysis, CNN-transformer hybrid model

## Abstract

Accurate assessment of N staging in patients with non-small cell lung cancer (NSCLC) is critical for the development of effective treatment plans, the optimization of therapeutic strategies, and the enhancement of patient survival rates. This study proposes a hybrid model based on 3D convolutional neural networks (CNNs) and transformers for predicting the N-staging and survival rates of NSCLC patients within the NSCLC radiogenomics and Nsclc-radiomics datasets. The model achieved accuracies of 0.805, 0.828, and 0.819 for the training, validation, and testing sets, respectively. By leveraging the strengths of CNNs in local feature extraction and the superior performance of transformers in global information modeling, the model significantly enhances predictive accuracy and efficacy. A comparative analysis with traditional CNN and transformer architectures demonstrates that the CNN-transformer hybrid model outperforms N-staging predictions. Furthermore, this study extracts the one-year survival rate as a feature and employs the Lasso–Cox model for survival predictions at various time intervals (1, 3, 5, and 7 years), with all survival prediction *p*-values being less than 0.05, illustrating the time-dependent nature of survival analysis. The application of time-dependent ROC curves further validates the model’s accuracy and reliability for survival predictions. Overall, this research provides innovative methodologies and new insights for the early diagnosis and prognostic evaluation of NSCLC.

## 1. Introduction

Lung cancer remains a malignant tumor with high incidence and mortality rates worldwide, drawing significant concern due to its severity and urgency [1]. Non-small cell lung cancer (NSCLC) constitutes the majority of lung cancer cases, accounting for 80% to 85% of all instances, leading to approximately 1.6 million deaths annually [2,3]. This presents a substantial challenge to public health. The eighth edition of the American Joint Committee on Cancer (AJCC) lung cancer staging system is a widely used tool, classifying NSCLC based on tumor size (T), lymph node involvement (N), and distant metastasis (M) [4]. This staging system provides clinicians with a fundamental description of the severity of lung cancer, serving as a critical reference for treatment planning and prognosis evaluation. The treatment strategy and prognosis of NSCLC are highly dependent on its staging system, especially the N staging, which is crucial for guiding treatment decisions and predicting patient survival [5].

N staging is divided into several levels based on the extent of lymph node involvement [4]. N0 indicates no lymph node metastasis. N1 represents hilar lymph node involvement without extending beyond the hilar nodes. N2 indicates regional (extrapulmonary) lymph node involvement. N3 denotes lymph node involvement that has spread to distant (remote) lymph nodes, potentially involving contralateral intrapulmonary or extrapulmonary lymph nodes. As an integral part of lung cancer diagnosis, N staging significantly impacts the formulation of personalized treatment plans. For example, patients with N0 staging might only require surgical resection, while those with N1 staging and beyond might need a combination of radiotherapy, chemotherapy, or immunotherapy [6]. Notably, approximately 30% to 40% of NSCLC patients experience lymph node metastasis during the course of the disease, which can not only lead to disease progression and deterioration but also cause a series of unpredictable complications, thereby significantly affecting treatment outcomes and survival rates [7]. Therefore, accurately assessing N staging is of paramount importance for developing effective treatment plans, optimizing therapeutic strategies, and improving patient survival rates.

Current methods for N staging for NSCLC mainly depend on imaging techniques (such as CT and PET-CT) and pathological analysis. Pathological methods, including tissue biopsy and molecular biology techniques, provide insights into tumor characteristics but are invasive and limited by sample acquisition [8,9,10,11]. Imaging techniques like CT and PET-CT offer anatomical and metabolic information that is crucial for staging, though they have limitations in detecting small or low-activity lesions and rely on physician expertise [12,13,14,15]. Therefore, there is a pressing need for more accurate, non-invasive techniques to improve N-staging accuracy and minimize human error.

In recent years, deep learning and machine learning have been widely applied in cancer-staging research. In traditional machine learning, Chen et al. [16] predicted STAS in stage-I lung adenocarcinoma using radiomic features, selecting five key features to construct a naive Bayes model with an AUC of 0.69, validating its application value in STAS prediction. Gu et al. [17] analyzed the CT images and clinical data of T1N0M0 lung adenocarcinoma patients, combining texture and clinical features to predict lymph node metastasis through a logistic regression model, achieving an AUC of 0.808 in the validation set, aiding in treatment planning. Parmar et al. [18] extracted quantitative features from CT images of 422 NSCLC patients, identifying 11 stable lung cancer-specific radiomic feature clusters, with external validation showing high stability and reliability, although the feature-extraction process was unclear. Parmer et al. [19] extracted 440 radiomic features from CT images of 464 NSCLC patients, evaluating the performance of various feature-selection and classification methods in predicting overall survival, with Wilcoxon feature selection and random forest classifier performing best. Janik et al. [20] constructed machine-learning models for early-stage NSCLC patient data, achieving accuracies of 0.76 and 0.68 in the prediction of recurrence for tabular and graph data models, respectively. Moon et al. [21] used PCA to extract features from postoperative lung tumor resection patient data, including radiomic features and clinical data, with the PCA-SVM model achieving the highest accuracy (ACC of 0.77) in the prediction of recurrence risk. Kim et al. [22] studied lymph node residuals in stage-IIIa NSCLC patients after neoadjuvant CCRT treatment, with the logistic regression model predicting metastasis with an AUC of 0.77. Gu-Wei Ji et al. [23] applied SVM and LASSO algorithms to establish radiomics nomograms, analyzing CT images of bile duct cancer patients to predict lymph node metastasis, with AUCs of 0.81 and 0.80, respectively. Cong et al. [24] developed a model combining clinical parameters and radiomic features to predict lymph node metastasis in stage-IA NSCLC patients using LASSO for feature selection and achieving an AUC of 0.86. Chen et al. [25] studied a radiomics nomogram that combines intratumoral and peritumoral features to predict lymph node metastasis and overall survival (OS) in clinical stage-IA non-small cell lung cancer. They selected 199 cases and defined four volumes of interest, using manually designed feature extractors. They developed a nomogram combining optimal radiomics features and clinical predictive factors with the Lasso–Cox method. The experiments demonstrated that GPTV6 (tumor and 6mm peritumoral volume) radiomics outperformed GTV, GPTV3, and GPTV9 radiomics in the training (area under the curve [AUC], 0.81), internal validation (AUC, 0.79), and external validation cohorts (AUC, 0.71).

In NSCLC prediction tasks, traditional machine-learning methods face challenges, such as reliance on manual feature extraction, which is time-consuming and may miss important data. Limited data also restricts model generalization, leading to inconsistent performance across datasets. Furthermore, balancing model complexity with interpretability is difficult, particularly in the medical field, where transparency in decision-making is crucial.

In terms of deep-learning methods, Wang et al. [26] utilized BP-ANN to extract features from the PET/CT images of NSCLC patients and classify them, achieving an ACC of 0.81–0.85 and an AUC of 0.87–0.92 in 10-fold cross-validation. Zhang et al. [27] used multi-view radiomics and ResNet18 to establish a model predicting N2 lymph node metastasis in clinical stage I-II NSCLC patients, with an AUC of 0.83. Zhong et al. [28] developed a ResNet152 deep-learning model for predicting N2 lymph node metastasis and prognostic stratification in stage-I NSCLC, achieving AUCs of 0.82, 0.81, and 0.81 in internal, external, and prospective test cohorts, respectively. Ouyang et al. [29] constructed a deep-learning model to predict occult lymph node metastasis (OLM) in clinically lymph node-negative (cN0) lung adenocarcinoma patients, with an AUC of 0.81 in internal validation and 0.87 in prospective testing. Sibille et al. [30] retrospectively analyzed the 18F-FDG PET/CT images of 629 lung cancer and lymphoma patients, evaluating the performance of a deep CNN in lesion localization and classification, with the DenseNet model excelling in identifying suspicious high-metabolic lesions. Zhao et al. [31] proposed a cross-modal 3D neural network model, DensePriNet, combining preoperative CT images and clinical prior knowledge to improve the accuracy of predicting lymph node metastasis in clinical T1-stage lung adenocarcinoma patients, with a test AUC of 0.926. Noam Tau et al. [32] used DenseNet to analyze 18F-FDG PET images of newly diagnosed NSCLC patients to predict the risk of lymph node and distant metastasis, achieving an AUC of 0.80 for lymph node metastasis prediction. Mu et al. [33] used ResNet18 to predict the malignancy and metastasis of solid lung nodules in CT images of primary lesions at initial diagnosis, with the deep-learning system achieving AUCs of 0.8037 and 0.8644 for malignancy and metastasis prediction, respectively. Lian et al. [34] developed a population graph model combining Transformer-based radiomics and clinical features to predict OS and RFS in early-stage NSCLC. Using 1,705 stage I-II patients and 127 for external validation, the model achieved OS AUCs of 0.785 (internal) and 0.695 (external) and RFS AUCs of 0.726 and 0.700.

In lung cancer staging and survival prediction, convolutional neural networks (CNNs) are widely used due to their strong ability to capture local features and retain spatial information, effectively analyzing tumor characteristics such as size, shape, and texture in CT images. However, CNNs are limited in capturing global contextual information, which is essential for understanding the complex relationship between tumors and surrounding tissues.

Transformer architectures, known for their global context comprehension and efficient parallel processing, excel in capturing long-range dependencies and tumor progression patterns. Yet, pure transformer models may struggle with fine-grained local details and require significant data and computational resources, limiting their direct application in medical imaging.

It is worth noting that, in other medical image-processing tasks, such as lesion classification [35,36,37], object detection [38,39], and segmentation [40,41], an increasing number of studies are adopting a hybrid approach that combines CNN and transformer architectures. The hybrid model capitalizes on CNNs’ local feature extraction and transformers’ global understanding, offering flexibility, scalability, and improved accuracy in predictions. This integration provides a more comprehensive and personalized diagnostic approach, addressing both tumor features and overall patient condition.

Based on the above analysis, we have decided to integrate CNN and transformer architectures in the task of lung cancer staging and survival prediction. This decision is guided by several considerations. First, a hybrid model can combine the local feature-extraction capabilities of CNNs with the global contextual understanding of transformers, thereby enhancing the accuracy and robustness of predictions. Second, the hybrid model offers greater flexibility and scalability, allowing us to adjust the ratio and configuration of CNNs and transformers according to specific task requirements to optimize model performance. Finally, the hybrid model addresses the practical need in lung cancer diagnosis to consider both local tumor features and the patient’s overall condition, providing more comprehensive, accurate, and personalized diagnostic and predictive results.

Thus, combining CNNs and transformers represents a promising solution for complex medical imaging tasks like lung cancer staging and survival prediction.

## 2. Model Description

**Figure 1:**
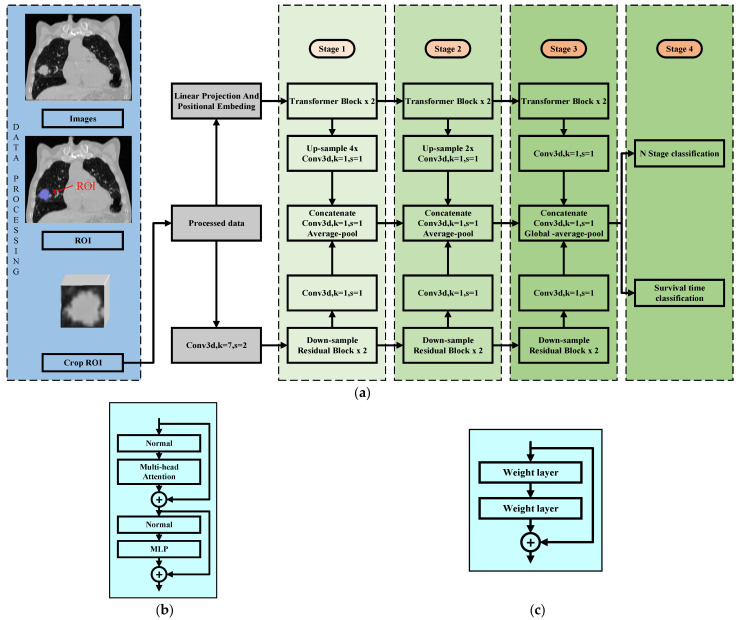
The complete information processing flow of the HCT Model. (**a**) HCT Model Structure. (**b**) The structure of transformer block. (**c**) The structure of residual block.

The workflow for predicting non-small cell lung cancer (NSCLC) N staging is shown in Figure 1. The proposed HCT model consists of three parts, namely the transformer feature-extraction block, the ResNet feature-extraction block, and the feature fusion module.

The transformer architecture, originally successful in NLP due to its self-attention mechanism that captures sequence dependencies, is used in the HCT model to capture the global context in 3D imaging data. It employs a multi-head self-attention mechanism to process each image region, capturing the interactions between different areas. This mechanism is shown in Equation (1).

(1) $$\left\{\begin{array}{l} Attention_{i}(Q_{i},K_{i},V_{i})=Soft\max(\frac{Q_{i}\times K_{i}^{T}}{\sqrt{d}})\times V_{i}\\ MultiHead=Concat(Attention_{1},\ldots Attention_{i})\\ Output=Input+FFN(MultiHead) \end{array}\right.$$

where, *Q*, *K*, and *V* represent the query, key, and value, respectively, and *d* is the dimensionality of the keys. *MultiHead* represents the concatenated multi-head attention, *Concat* denotes the concatenation operation, *FFN* stands for feedforward neural network, *Input* refers to the input features, and *Output* refers to the output features.

ResNet (residual network) is a major advancement in deep learning, solving the vanishing gradient issue in deep networks through residual connections. In the HCT model, the ResNet module extracts local features from 3D imaging data using convolutional layers and residual connections, allowing the network to capture detailed image information. The residual structure of the ResNet module is defined by Equation (2).

(2) $$y_{1}=h(x_{1})+F(x_{l},\left\{W_{i}\right\})$$

where, $x_{l}$ represents the input to the *l*-th layer, $h(x_{1})$ denotes the output of the convolutional layer, and $F(x_{l},\left\{W_{i}\right\})$ signifies the weights of the convolutional layer. The identity mapping is represented by $\left\{W_{i}\right\}$.

The feature fusion module is the core of the HCT model, combining features from both the transformer and ResNet to create a more comprehensive feature representation. It uses convolutional layers, downsampling, and other techniques to merge features across different scales and levels. The fusion process is described in Equation (3).

(3) F =Conv(Concat(FTransformer ,FResNet ))

where $F_{Transformer}$ and FResNet  represent the features extracted by the transformer and ResNet modules, respectively.

In hybrid models, CNNs and transformers complement each other by capturing local details and global context. The feature fusion module, the key to integrating this information, is critical for model performance. It consists of 1 × 1 convolution and feature concatenation. The 1 × 1 convolution adjusts the output channels, enabling a seamless concatenation of features from both networks while extracting useful information.

Feature concatenation offers clear advantages over addition by fully retaining all information from CNNs and transformers, avoiding bias or loss. This improves classification accuracy and model robustness, particularly in NSCLC staging and survival analysis, allowing the model to capture tumor patterns and relationships more precisely. Consequently, feature concatenation enhances model performance and generalization and excels in these complex tasks.

The processing workflow starts with data preprocessing, converting raw lung cancer images into regions of interest (ROI) and performing 3D cuboid cropping to focus on key diagnostic areas, reducing data size while retaining critical information. Next is the feature extraction, where stacked transformer and ResNet blocks progressively extract image features. Transformers capture global features and long-range dependencies, crucial for understanding the image structure and context. Conversely, the ResNet blocks focus on extracting local features and capturing detailed information within the image, which are essential for identifying small lesion areas. After feature extraction, the model uses a 1×1 convolutional layer to fuse features from different transformer blocks and ResNet blocks. The fused feature maps are combined into a comprehensive feature set through a multi-layer, multi-scale fusion process, enhancing precision and robustness in lung cancer detection. A custom ROI classifier then analyzes global and local information to make accurate classification decisions, offering reliable diagnostic support.

## 3. Data Preprocessing and Experimental Parameter Settings

The NSCLC-Radiomics dataset [42] includes imaging data from 422 NSCLC patients across three types, namely adenocarcinoma (52 cases), large cell carcinoma (114 cases), and squamous cell carcinoma (152 cases). The NSCLC Radiogenomics dataset [43] focuses on the radiogenomics of adenocarcinoma (112 cases) and squamous cell carcinoma (29 cases), with 211 subjects in total. Training and validation sets were sourced from Maastricht University Medical Center, and the validation set came from Stanford University and the Palo Alto, VA, USA, representing different ethnicities and countries. After excluding incomplete cases, 380 patients from the NSCLC-Radiomics dataset and 133 from the NSCLC Radiogenomics dataset were included. Seventy percent of the NSCLC-Radiomics dataset was used for training, with the rest for internal validation, while the NSCLC Radiogenomics dataset served as an external test set. This diverse data ensured robust validation, and chi-square tests confirmed no statistical differences between the training and validation sets (*p* > 0.05), ensuring an unbiased split, as shown in Table 1.

During CT data preprocessing, images are resampled to a 1 mm^3^ voxel size, with the HU values standardized between −600 and 600 using a window width of 1200 and center of zero. This ensures consistency across scans and improves model performance. Slices with the largest VOI are selected, and a minimal bounding cuboid is extracted to reduce background noise. Z-score normalization is applied to standardize intensity distributions, and these images are used for deep-learning input. In training, random cropping and flipping enhance generalization, while testing only uses standardization for consistency. The main goal of these strategies is to improve robustness and accuracy across varying scans and datasets.

During the model-training phase, cropped sub-region images are resized to 1×1×1 using nearest-neighbor interpolation to fit the model’s input dimensions. Given the limited amount of image data, the learning rate is carefully chosen to enhance the model’s generalization performance. A cosine annealing learning rate algorithm is employed, which is defined by the following formula:

(4) $$\eta_{t}=\eta_{\min}^{i}+\frac{1}{2}(\eta_{\max}^{i}-\eta_{\min}^{i})(1+\cos(\frac{T_{cur}}{T_{i}}\pi ))$$

where $\eta_{\min}^{i}=0$ is the minimum learning rate, $\eta_{\max}^{i}=0.01$ is the maximum learning rate, and $T_{i}=96$ represents the number of cycles during the iterative training process. This study uses stochastic gradient descent (SGD) as the optimizer and softmax cross-entropy as the loss function, aiming to optimize the training performance and ensure stable, robust learning.

The analysis uses Python 3.7, statsmodels 0.13.2, and scikit-learn 1.0.2 for deep-learning model development. Training is powered by an NVIDIA 4090 GPU with MONAI and PyTorch frameworks. The NVIDIA 4090 GPU is manufactured by NVIDIA Corporation in Santa Clara, CA, USA. Features from the penultimate model layer are selected using the Lasso–Cox method for survival analysis. The study applies the Cox proportional hazards model with L2 regularization and Kaplan–Meier analysis, classifying the samples by predicted hazard ratios and evaluating stratification via the multivariate log-rank test.

## 4. Experimental Metrics Explanation

To assess the performance of the proposed model for predicting the N staging of non-small cell lung cancer (NSCLC), the following metrics are introduced:

accuracy: the proportion of correctly classified instances (both true positives and true negatives) out of the total number of instances. It is given by

(5) $$Accuracy=\frac{TP+TN}{FP+FN+TP+TN};$$

sensitivity: the proportion of actual positive cases that are correctly identified by the model. It is given by:

(6) $$Sensitivity=\frac{TP}{TP+FN};$$

specificity: the proportion of actual negative cases that are correctly identified by the model. It is given by:

(7) $$Specificity=\frac{TN}{FP+TN\;}.$$

In these formulas, true positive (TP) represents cases where the model correctly predicts the positive class. True negative (TN) represents cases where the model correctly predicts the negative class. False positive (FP) represents cases where the model incorrectly predicts the positive class for samples that are actually negative. False negative (FN) represents cases where the model incorrectly predicts the negative class for samples that are actually positive.

A 95% confidence interval (95% CI) provides a range within which the true value of the metric is expected to fall with 95% confidence. It is computed for each metric to provide an estimate of the variability and reliability of the metric. For example, for accuracy, the confidence interval can be calculated using statistical methods or bootstrapping techniques.

To calculate the 0.95 confidence interval for the accuracy of a model, the following expression is used:

(8) CI=p^±Zα2×p^(1−p^)n

In this context, $\hat{p}$ is the estimated accuracy of the sample. Zα2 is the critical value from the standard normal distribution corresponding to the upper α2 quantile of the confidence level. n is the sample size. $\hat{p}(1-\hat{p})$ is the variance estimate of the sample accuracy. This formula calculates the confidence interval for accuracy, where there is a 95% probability that the true value of the population accuracy falls within this interval.

AUC, or the area under the curve, represents the area under the ROC (receiver operating characteristic) curve and provides a comprehensive assessment of the model’s predictive performance across different thresholds. The ROC curve plots the relationship between the true-positive rate (TPR, equivalent to Sensitivity) and the false-positive rate (FPR, equivalent to 1-Specificity). The AUC measures the overall performance of the model across the entire ROC curve. The AUC for that class can be represented as:

(9) AUCc=∫01Sensitivityc((1-Specificity)−1(t))cdt

In this context, (1-Specificity)−1(t)c denotes the inverse function of ${\left(1-Specificity\right)}_{c}$. It is used to calculate the false-positive rate given a specific true-positive rate. The trapezoidal rule is typically used for numerical integration to approximate this area.

The micro-AUC can be calculated by taking the weighted average of the AUCs for each class. If $n_{0},n_{1},n_{2},n_{3}$ represents the number of samples for each class, then the micro-AUC can be computed as follows:

(10) $$microAUC=\frac{\sum_{c=0}^{3}\;n_{c}\;\;\cdot AUC_{c}}{\sum_{c=0}^{3}\;n_{c}}$$

## 5. Experimental Results

### 5.1. N Stage Prediction and Survival Analysis

To evaluate the effectiveness of the HCT model in diagnosing N staging of non-small cell lung cancer (NSCLC), this study conducted a comprehensive performance analysis of five different deep-learning models. These models include the HCT model, DenseNet121, ResNet50, ShuffleNet, and ViT. Among them, DenseNet121, ResNet50, and ShuffleNet are based on convolutional neural network (CNN) architectures, while ViT is based on transformer architecture. HCT combines both CNN and transformer architectures. To ensure fairness in the model comparisons, all models compared in Table 2 used the same optimizer and were trained for the same number of epochs. During the evaluation, we used performance metrics to assess the models, including accuracy (ACC), sensitivity, and specificity, with results presented in Table 2. These metrics help us gain a comprehensive understanding of each model’s performance in the diagnosis of N staging in NSCLC patients.

**Figure 2:**
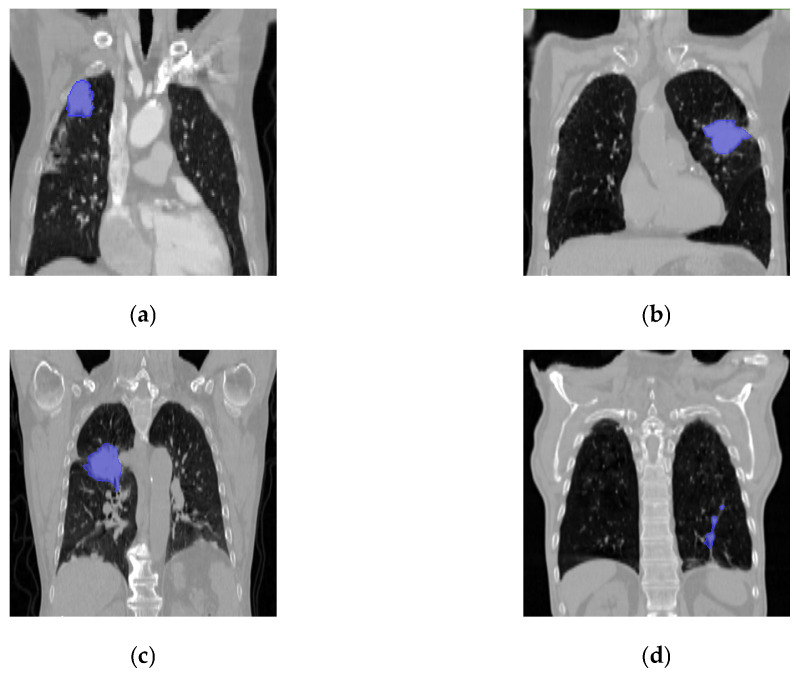
Examples of cases correctly and incorrectly classified by the model, with the primary tumor marked in blue. (**a**) Cases in N0 stage misclassified as N1 stage. (**b**) Cases in N1 stage correctly identified. (**c**) Cases in N2 stage correctly identified. (**d**) Cases in N3 stage misclassified as N2 stage.

In the precision-medicine task of N staging for non-small cell lung cancer (NSCLC), evaluating the performance of different models during training, validation, and testing phases is crucial, as it directly reflects the overall predictive effectiveness of the models. Notably, the HCT model demonstrated an exceptional accuracy of over 0.8 in all three sets, significantly surpassing other comparative models, which strongly supports the HCT model’s superior capability in accurately determining lung cancer staging in patients. Furthermore, the specificity metric reveals that the HCT model has high precision in effectively distinguishing between healthy individuals and NSCLC patients, with the specificity values staying above 0.8 across all stages, markedly outperforming other models. This underscores the model’s high accuracy in identifying disease status. Additionally, the HCT model’s positive predictive value in identifying specific N stages is also significantly better than that of other models, indicating its advantage for reducing misdiagnosis. Examples of cases correctly and incorrectly classified by the HCT model are shown in Figure 2.

The HCT model exhibits a significant advantage in handling 3D medical imaging data due to its innovative structural fusion, combining the strengths of convolutional neural networks (CNNs) and transformers. Traditional CNN architectures, such as DenseNet121, ResNet50, and ShuffleNet, excel in extracting local features but struggle with the complex spatial structures and contextual relationships in 3D data, requiring additional adjustments to accommodate the increased data dimensions. In contrast, Transformer-based models like ViT can naturally capture long-range dependencies through self-attention mechanisms, which are crucial for understanding global information. However, they are less efficient in the initial stages of extracting local image features compared to CNNs. The HCT model cleverly integrates both approaches, allowing CNNs to extract rich local features from within the image and leveraging transformers to use these features to build global dependencies through self-attention mechanisms. This complementary approach not only overcomes the limitations of each individual method but also enhances the model’s ability to comprehensively understand and analyze complex 3D imaging data, providing more precise and comprehensive information support for medical diagnosis and analysis.

For the HCT model, the AUC scores consistently exceed 0.8 across all dataset segments, indicating strong discriminative power. Specifically, the AUC is 0.837 for the training cohort, 0.813 for the validation cohort, and 0.816 for the testing cohort. These results demonstrate robust performance across different samples. In contrast, DenseNet121 achieves an AUC of 0.862 for the training cohort, but this significantly drops in the validation (0.741) and testing cohorts (0.732), suggesting potential overfitting to the training data. On the other hand, ShuffleNet shows moderate AUC scores, with a maximum of 0.784 for the training cohort and a minimum of 0.637 for the testing cohort. ResNet50 exhibits moderate AUC scores as well, with a highest of 0.798 for the training cohort and a lowest of 0.722 for the testing cohort. ViT also shows moderate AUC scores, with a highest of 0.794 for the training cohort and a lowest of 0.756 for the testing cohort. Compared to DenseNet121, ShuffleNet, ResNet50, and ViT, the HCT model demonstrates superior generalization and stability across different datasets. As illustrated in Table 2, the consistently high AUC values above 0.8 for the training, validation, and testing cohorts highlight the robustness and reliability of HCT. In contrast, the significant variability in the AUC scores for DenseNet121, ShuffleNet, ResNet50, and ViT suggests that these models may perform less effectively when applied to different datasets. DenseNet121 and ResNet50 effectively capture complex data features with their deep structures and large parameters but are prone to overfitting the training data and may face gradient vanishing or explosion issues, impacting stability and convergence. ShuffleNet enhances efficiency through grouped convolutions and channel shuffling but limits the feature communication between groups, affecting the overall feature extraction. Although ViT captures long-range dependencies, it may overlook local information in medical image processing. Therefore, due to its consistent performance and prediction accuracy, HCT emerges as a more desirable choice.

In addition, Table 3 presents a summary of the latest research on classification models for NSCLC, provided for reference and comparative analysis.

**Figure 3:**
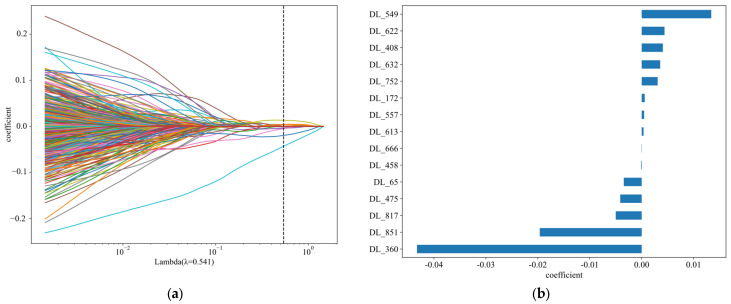
Results of the Lasso–Cox Algorithm for feature selection in N-staging prediction. (**a**) Lasso–Cox Result. (**b**) Features filtered by Lasso algorithm.

Further, the study employed the Lasso–Cox method for feature selection and analyzed the impact of the selected features on patient survival time. The Lasso–Cox method successfully identified and retained the 15 most influential features contributing to the model’s predictive performance, as illustrated in Figure 3. With increasing regularization parameters, the coefficients of most features approached zero. The optimal regularization parameter value was determined to be 0.541, at which point, the coefficients of the 15 selected features were found to be significant.

**Figure 4:**
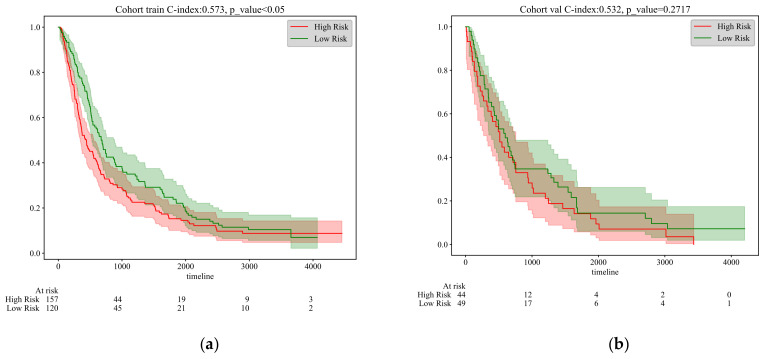

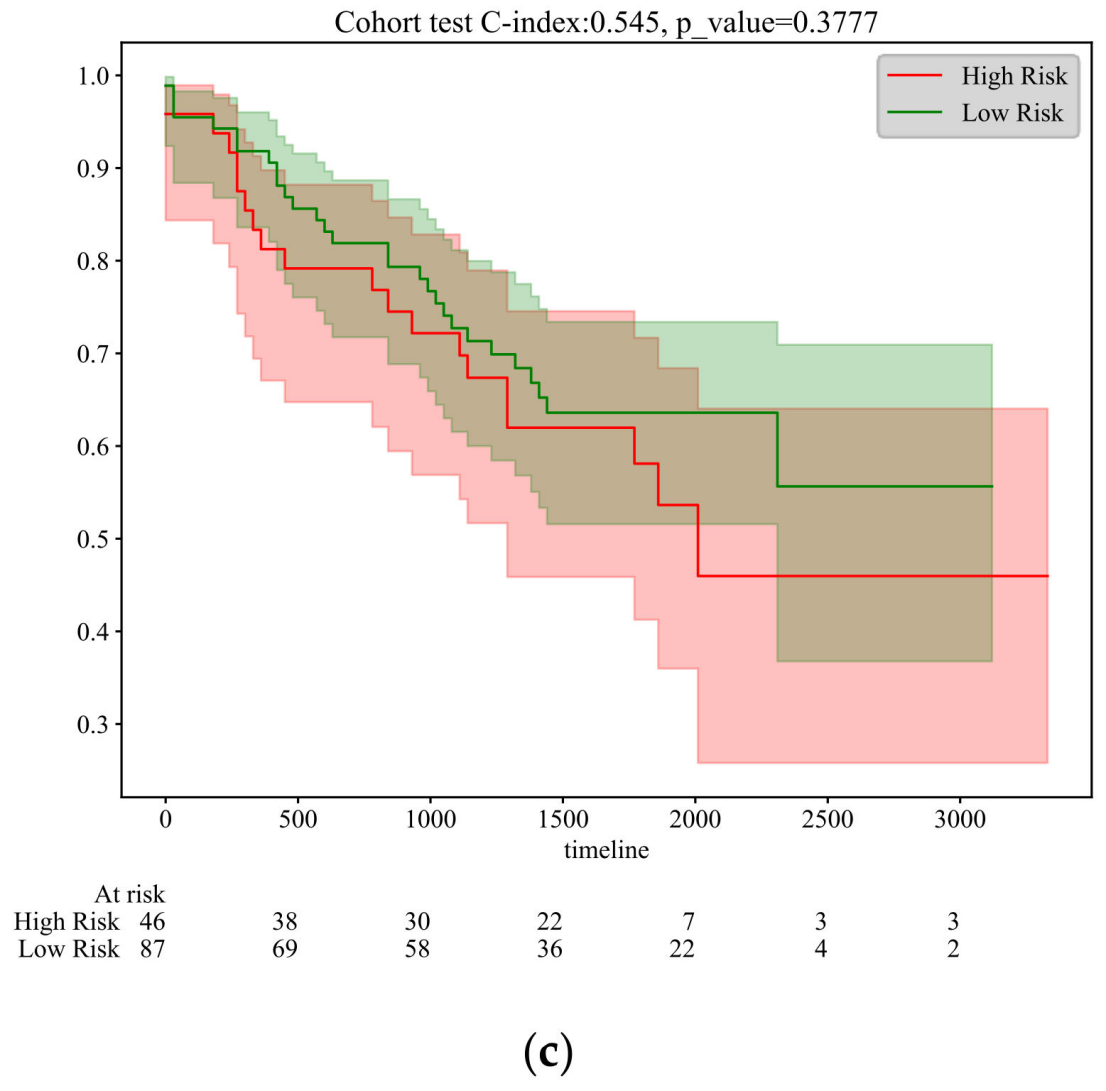
Kaplan–Meier survival curves of different cohorts at N stages. (**a**) Training cohort. (**b**) Validation cohort. (**c**) Test cohort.

Figure 4 presents the results of survival analysis for NSCLC patients based on the N-staging classification features extracted by the model, highlighting the performance of the model based on risk grouping (high risk vs. low risk). In the training set, the survival curves for the two groups are significantly separated, with the high-risk group having a lower survival rate than the low-risk group, indicating effective risk differentiation by the model on the training data. However, although the C-index is 0.573 (*p* < 0.05), and statistically significant, it is close to 0.5, suggesting a limited discriminative ability of the model. Moving to the validation set, the confidence intervals of the survival curves widely overlap, with the C-index dropping to 0.532 (*p* < 0.2172). Despite still being statistically significant, this indicates further limitations to the model’s discriminative ability. The performance on the test set is even poorer, with a C-index of 0.545 and a *p*-value rising to 0.3777 (*p* > 0.05), losing statistical significance, and approaching the level of random prediction, strongly suggesting a limited generalizability of the model on unseen data.

In summary, although the model demonstrates some distinguishing efficacy within the training set, its performance significantly declines in the validation and test sets, particularly failing to achieve statistically significant predictive ability on the test set. This reflects the model’s limitations in external validation and the need for further optimization. Additionally, the C-index values approaching the threshold indicate that the model’s predictive accuracy needs improvement, potentially requiring the inclusion of more relevant features to enhance its predictive precision and robustness.

### 5.2. Survival Time Prediction

In the deep-learning study focused on the N staging of non-small cell lung cancer (NSCLC) described above, we successfully achieved AUC values exceeding 0.80 at different staging phases. Subsequently, we conducted a further analysis of survival time prediction using the features extracted for N staging. However, in both the internal validation and external test sets, we observed that the *p*-values were greater than 0.05, indicating that the extracted features did not show significant statistical significance in the prediction of survival time. Therefore, we infer that these features might not be sufficient for accurate survival time prediction. To address this issue, we turned to extracting time-related features and incorporated a loss function for 1-year survival time into our experimental design.

We trained a time-dependent binary classification model to predict 1-year survival rates. The HCT model demonstrated outstanding performance in the deep-learning-based NSCLC N staging experiments, maintaining significant stability in its performance metrics. As a result, we selected the HCT model for the time-dependent binary classification task. The HCT model showed high and consistent AUC values across different datasets (training, test, and validation), proving its strong ability to handle data variability and extract key features. Given its overall excellence, we chose the HCT model for feature extraction and constructed a survival analysis model. Table 4 presents the corresponding performance metrics.

**Figure 5:**
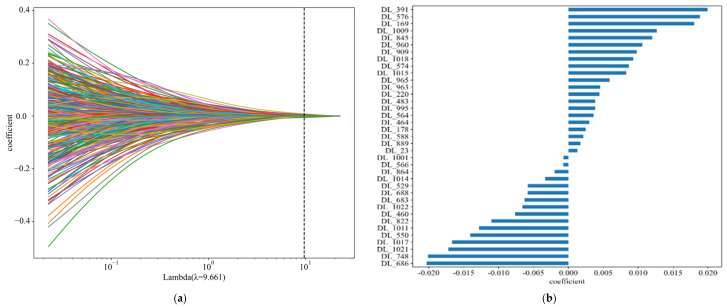
Lasso–Cox result chart for one-year rate period. (**a**) Lasso-Cox result. (**b**) Features selected by Lasso-cox algorithm.

In the study of 1-year survival prediction for non-small cell lung cancer (NSCLC) patients using the Lasso–Cox regression method, we achieved significant progress based on the HCT model. Through meticulous statistical analysis, we successfully identified 38 features that contribute most significantly to the model’s predictive performance. This number far exceeds the 15 features identified by the HCT model for NSCLC N staging, thereby significantly expanding the feature dimensions of the prediction model. The results are illustrated in Figure 5.

Figure 5a reflects the trend in which the correlation coefficients of the features obtained by the deep-learning feature extractor approach zero as the regularization parameter lambda increases in the Lasso–Cox algorithm. In the figure, when lambda is set to 9.661, 38 retained features have a significant impact on survival, while the discarded features help prevent overfitting due to an excessive number of variables. Figure 5b shows these 38 retained features and lists their importance weights in the model. The larger the absolute value of the coefficient, the greater its impact on patient survival. This approach effectively enhanced the model’s sensitivity to variations in survival time. To visually present the importance of these features, we utilized graphical representation, displaying the coefficients of each feature in ascending order. This visual representation not only improved the interpretability of the results but also allowed researchers to easily understand the contribution of each feature to the prediction model. It provides a solid data foundation for further clinical decision support and the optimization of treatment strategies.

**Figure 6:**
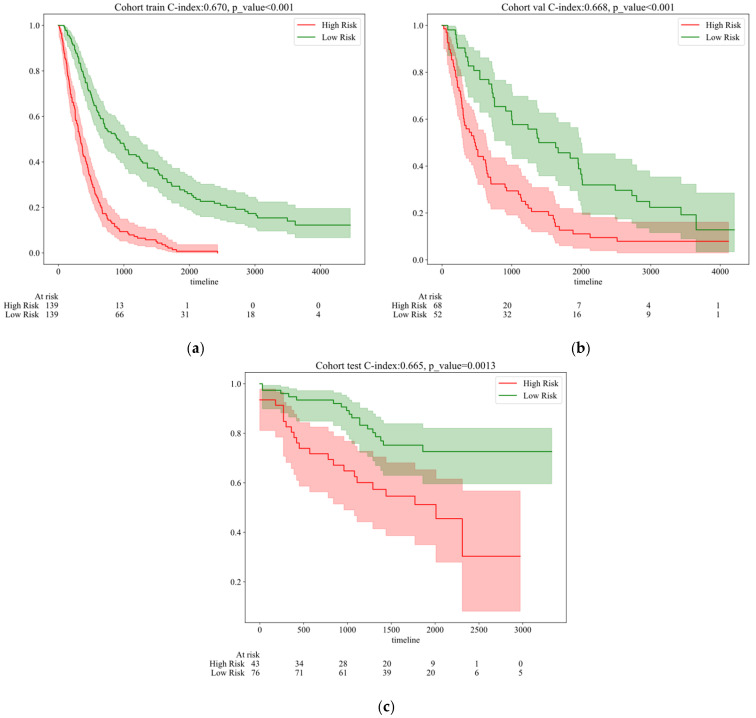
Kaplan–Meier survival curves of different cohorts by features extracted from the one-year survival rate. (**a**) Training cohort. (**b**) Validation cohort. (**c**) Test cohort.

Through training, the model successfully identified significant survival probability differences between the two groups, as visually demonstrated by the Kaplan–Meier survival curves. As shown in Figure 6, the survival rate of the high-risk group significantly declines over time. The model achieved a C-index of 0.670 on the training set, with a very low *p*-value (<0.001), strongly confirming its predictive performance and the statistical significance of the group differences. Further evaluation of the validation set confirmed the model’s generalization capability, with a C-index of 0.668 and a *p*-value still significantly below 0.001. This indicates that the model performs effectively not only on the training data but also maintains high discrimination and predictive accuracy on an independent-validation dataset. Finally, the results from the test set (C-index = 0.665, *p* = 0.0013) reinforced the model’s generalization performance on unseen data, effectively differentiating survival probabilities among risk groups and confirming the statistical significance of this differentiation. In the test set, the increase in the *p*-value may be due to the validation data being collected at different institutions using various pieces of equipment. Additionally, the validation set includes diverse ethnicities and age groups, such as Asian, Caucasian, and African populations.

Table 5 presents the results of survival-dependent features extracted by the model for predicting survival time. From the data in Table 5, it is evident that, as the prediction survival time increases, the model’s performance in terms of sensitivity and specificity also varies. Sensitivity and specificity are two critical metrics for evaluating model performance, measuring the accuracy of the model in the identification of positive cases and negative cases, respectively. In the training cohort, sensitivity fluctuates with an increasing prediction time, while specificity shows a significant improvement. This indicates that, over time, the model becomes more adept at accurately identifying true survivors in the training cohort.

Overall, as the predicted survival time increases, the model shows an increase in specificity in the training cohort, while sensitivity and specificity exhibit varying trends in the validation and test cohorts. These variations reflect differences in the model’s predictive capabilities at different survival time points and its performance across different datasets (training, validation, and test).

**Figure 7:**
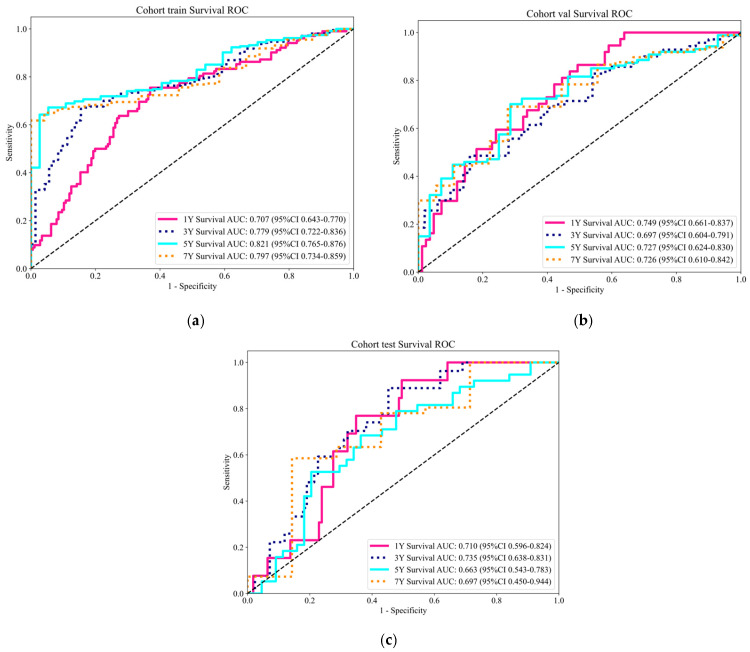
ROC Curves for time-dependent analysis. (**a**) Training cohort. (**b**) Validation cohort. (**c**) Test cohort.

Figure 7 presents the ROC curves for time-dependent analysis. Combined with Table 5 and Figure 7, the AUC (area under the curve) results demonstrate the model’s ability to predict survival at different time points across three cohorts (training, validation, and test). In the training cohort, the AUC values increase with the length of the predicted survival time, rising from 0.707 for 1-year survival to 0.797 for 7-year survival. In the validation cohort, the AUC values are relatively lower, peaking at 0.749 for 1-year survival and dropping to a low of 0.697 for 3-year survival. In the test cohort, the AUC values show more variability, with the highest value being 0.735 for 3-year survival and the lowest being 0.663 for 5-year survival. These variations highlight how the model’s prediction accuracy changes based on the length of the predicted survival time and the cohort being evaluated.

An analysis of the AUC metrics at different time points and across different cohorts indicates that the model performs more robustly for long-term survival predictions in the training cohort, where the clinical data and model training are closely aligned. However, as the prediction window extends, AUC values in the validation and test cohorts generally decline or fluctuate. This suggests that the model may be overfitting to the training data or facing challenges when generalizing to new unseen data. This indicates the need for further model refinement and validation, particularly in real-world clinical settings, where diverse patient data and varying conditions can significantly impact model performance. Enhancing the robustness and reliability of predictions is essential to ensure the model’s effectiveness across different scenarios and patient populations.

## 6. Conclusions

The hybrid model proposed in this study integrates the advantages of 3D convolutional neural networks (CNNs) and transformers, demonstrating remarkable performance in N staging and survival prediction for non-small cell lung cancer (NSCLC). This model leverages the multi-layer convolutional and pooling operations of 3D CNNs to accurately capture subtle structural features within CT scan images, providing a solid foundation for disease staging. Simultaneously, the self-attention mechanism of the transformer endows the model with robust capabilities for global information integration and long-range dependency modeling, effectively enhancing the overall contextual understanding and predictive performance. This ingenious combination not only addresses the limitations of single CNNs in the processing of global information but also compensates for the challenges transformers face when directly handling image data, resulting in significant improvements in both prediction accuracy and efficiency. This opens up new avenues for the precise diagnosis of NSCLC. Moreover, the study delves into the time sensitivity of survival predictions by conducting predictive experiments at various time points and incorporating the one-year survival rate as a key feature, revealing a close relationship between survival predictions and temporal variations. The stable performance of time-dependent ROC curves substantiates the model’s exceptional capability in capturing dynamic changes in patient survival. This finding not only underscores the critical role of temporal factors in survival predictions but also equips clinicians with more precise and dynamic prognostic assessment tools. Additionally, the phenomenon of “time windows” identified in the research further elucidates key time points in disease progression, providing valuable insights for optimizing predictive models and enhancing accuracy, as well as offering significant references for the development of personalized treatment strategies for NSCLC.

However, this study has corresponding limitations, primarily manifested in four aspects. For the limitations of the dataset, although the mixed model demonstrates significant advantages over the existing dataset, the dataset itself may exhibit biases, such as regional differences in patient populations, the diversity of treatment methods, and inconsistencies in the data collection process. These factors may impact the model’s generalization capability. For computational cost limitations, the mixed model combines the spatial feature-extraction capabilities of CNNs with the global context modeling abilities of transformers, resulting in a need to process a large number of parameters and data during both training and inference. Particularly when handling high-resolution medical images or large-scale datasets, the computational demands of the mixed model increase dramatically, requiring substantial hardware resources (e.g., GPUs, TPUs). This not only raises the difficulty and time costs of model training but also limits its application in resource-constrained environments. For the limitations of feature interpretability in deep learning, while the mixed model can automatically learn and extract deep features from data, these features are often highly abstract and difficult to understand. Medical experts are more concerned with the biological significance behind the model’s predictions rather than merely mathematical or statistical patterns. However, due to the “black box” nature of the mixed model, its internal decision-making processes are challenging to intuitively interpret, which limits physicians’ trust and acceptance of the model’s predictions. Additionally, the lack of interpretability hinders scientists from deriving biologically meaningful insights with practical guidance from the model. For temporal dependency in survival prediction, although this study considers survival predictions at different time points, the model’s adaptability to dynamic factors, such as changes in patient conditions and treatment responses, requires further validation. For the validation of clinical applications, despite the encouraging experimental results, the practical effectiveness of the model in clinical applications still needs to be verified through large-scale, multi-center clinical trials.

In the future, the precise diagnosis of lung cancer can develop in the following directions.

For multimodal data fusion, by incorporating patient data from diverse regions and utilizing various treatment methods, alongside imaging, genomics, proteomics, and other multimodal data, a more comprehensive and representative dataset can be constructed to enhance the model’s generalization capability and accuracy.

For computational efficiency optimization, developing more efficient algorithms and training strategies, such as model pruning, quantization, and knowledge distillation, can reduce the number of model parameters and computational load. Additionally, leveraging distributed computing and edge computing technologies can improve training and inference speeds. The development of specialized hardware to accelerate specific types of computational tasks also represents an important direction.

For enhanced feature interpretability, researching and integrating more interpretable deep-learning architectures and techniques, such as attention mechanism visualization, rule-based model fusion, and concept activation vectors (CAV), can help reveal the internal decision-making processes of models and improve feature interpretability. Furthermore, incorporating knowledge from the medical field to construct biologically meaningful feature representations can facilitate better explanations of model predictions.

For real-time dynamic prediction, developing dynamic prediction models that can update patient information in real-time, adjusting predictions based on changes in patient conditions and treatment responses, is crucial. This necessitates models with strong adaptive capabilities and efficient computational power.

For large-scale clinical trials and clinical decision support systems, large-scale, multi-center clinical trials can evaluate the model’s stability and accuracy in various medical institutions and with various equipment conditions. Moreover, integrating the model into clinical decision support systems can provide physicians with AI-assisted diagnostic and prognostic evaluation tools based on big data, thereby enhancing the efficiency and quality of healthcare services.

For cross-disease expansion, after validating the effectiveness of this mixed model in the NSCLC domain, efforts can be made to extend its application to other types of cancer or disease areas to explore its universality and transferability. This would contribute to the further advancement of precision medicine and personalized treatment.

## Figures and Tables

**Table 1:** Baseline characteristics of our cohort.

Feature_Name		ALL	Train	Val	Test	*p* Value
Age		68.33 ± 9.63	67.89 ± 10.22	68.43 ± 9.24	69.23 ± 8.59	0.44
Gender	Female	154 (29.62)	90 (32.37)	32 (26.67)	32 (26.23)	0.335
	Male	366 (70.38)	188 (67.63)	88 (73.33)	90 (73.77)	

**Table 2:** Evaluation indicators for N staging using different deep-learning models.

Model	Acc	Micro-AUC	95% CI	Sensitivity	Specificity	Cohort
HCT	0.805	0.837	0.788–0.845	0.757	0.817	Train
HCT	0.828	0.813	0.750–0.854	0.724	0.835	Val
HCT	0.819	0.816	0.771–0.860	0.693	0.881	Test
DenseNet121	0.773	0.862	0.838–0.885	0.808	0.761	Train
DenseNet121	0.745	0.741	0.629–0.753	0.613	0.710	Val
DenseNet121	0.737	0.732	0.603–0.700	0.796	0.530	Test
ResNet50	0.786	0.798	0.753–0.832	0.728	0.721	Train
ResNet50	0.773	0.766	0.649–0.743	0.622	0.748	Val
ResNet50	0.792	0.722	0.673–0.739	0.686	0.617	Test
ShuffleNet	0.713	0.784	0.754–0.814	0.746	0.702	Train
ShuffleNet	0.644	0.687	0.625–0.748	0.663	0.638	Val
ShuffleNet	0.734	0.637	0.584–0.691	0.543	0.848	Test
VIT	0.783	0.794	0.734–0.824	0.716	0.792	Train
VIT	0.764	0.767	0.695–0.778	0.673	0.818	Val
VIT	0.799	0.754	0.716–0.821	0.651	0.837	Test

**Table 3:** A summary of the latest research on classification models for NSCLC.

References	Number of Samples	Tasks	Methods	Metrics
[17]	501	Lymph node metastasis in T1N0M0 stage lung adenocarcinoma patients	Logistic	AUC: 0.808
[18]	422	Staging status of non-small cell lung cancer patients	Clustering	AUC: 0.61 ± 0.01
[28]	140	N2 lymph node status in stage I-II NSCLC patients	ResNet18	AUC: 0.83
[30]	376	Occult lymph node metastasis in cN0 adenocarcinoma patients	Inception V3 (2D)	AUC: 0.81
[31]	629	Suspicious and non-suspicious states of lung cancer lesions	DenseNet	ACC: 0.981
[33]	264	Distant metastasis status in NSCLC patients	DenseNet	AUC: 0.65 ± 0.05
[34]	689	Malignancy degree of PNs in stage T1 lung cancer patients	ResNet18	AUC: 0.8037

**Table 4:** Evaluation indicators of HCT model under 1-year rate period.

Model Name	Acc	AUC	95% CI	Sensitivity	Specificity	Cohort
HCT	0.745	0.772	0.7156–0.8281	0.726	0.756	train
HCT	0.700	0.676	0.5711–0.7800	0.757	0.630	val
HCT	0.729	0.618	0.4401–0.7949	0.715	0.742	test

**Table 5:** Indicators for predicting 1-year, 3-year, 5-year, and 7-year survival risks based on different cohorts’ 1-year survival rates.

Survival	Accuracy	AUC	95% CI	Sensitivity	Specificity	Cohort
1Y Survival	0.773	0.707	0.6433–0.7702	0.745	0.831	Train
3Y Survival	0.808	0.779	0.7216–0.8362	0.762	0.845	Train
5Y Survival	0.823	0.821	0.7654–0.8765	0.768	0.946	Train
7Y Survival	0.748	0.797	0.7337–0.8594	0.714	1.000	Train
1Y Survival	0.763	0.749	0.6606–0.8373	0.758	0.806	Val
3Y Survival	0.785	0.697	0.6041–0.7908	0.771	0.840	Val
5Y Survival	0.796	0.727	0.6242–0.8298	0.790	0.814	Val
7Y Survival	0.785	0.726	0.6102–0.8422	0.780	0.822	Val
1Y Survival	0.767	0.710	0.5957–0.8242	0.756	0.805	Test
3Y Survival	0.774	0.735	0.6379–0.8313	0.772	0.848	Test
5Y Survival	0.759	0.663	0.5427–0.7826	0.766	0.795	Test
7Y Survival	0.714	0.697	0.4498–0.9440	0.701	0.757	Test

## Data Availability

The datasets generated and analyzed during the current study is are available in the [NSCLC Ra-diogenomics] repository at https://www.cancerimagingarchive.net/collection/nsclc-radiogenomics/. The access date is 23 March 2024. The datasets generated and analyzed during the current study are available in the [Nsclc-radiomics] repository at https://www.cancerimagingarchive.net/collection/nsclc-radiomics/. The access date is 18 February 2024.
